# BIRAFFE2, a multimodal dataset for emotion-based personalization in rich affective game environments

**DOI:** 10.1038/s41597-022-01402-6

**Published:** 2022-06-07

**Authors:** Krzysztof Kutt, Dominika Drążyk, Laura Żuchowska, Maciej Szelążek, Szymon Bobek, Grzegorz J. Nalepa

**Affiliations:** 1 Jagiellonian Human-Centered Artificial Intelligence Laboratory (JAHCAI) and Institute of Applied Computer Science, Jagiellonian University, Kraków, Poland; 2 Institute of Neuroscience, Université Catholique de Louvain, Louvain-la-Neuve, Belgium; 3 Department of Applied Computer Science, AGH University of Science and Technology, Kraków, Poland

**Keywords:** Human behaviour, Emotion, Information technology

## Abstract

Generic emotion prediction models based on physiological data developed in the field of affective computing apparently are not robust enough. To improve their effectiveness, one needs to personalize them to specific individuals and incorporate broader contextual information. To address the lack of relevant datasets, we propose the 2nd Study in Bio-Reactions and Faces for Emotion-based Personalization for AI Systems (BIRAFFE2) dataset. In addition to the classical procedure in the stimulus-appraisal paradigm, it also contains data from an affective gaming session in which a range of contextual data was collected from the game environment. This is complemented by accelerometer, ECG and EDA signals, participants’ facial expression data, together with personality and game engagement questionnaires. The dataset was collected on 102 participants. Its potential usefulness is presented by validating the correctness of the contextual data and indicating the relationships between personality and participants’ emotions and between personality and physiological signals.

## Abstract (summary)

**Table:** 

Measurement(s)	personality trait • gaming experience • game experience • Electrocardiography • Galvanic Skin Response • Accelerometer • Gyroscope • In-Game Activity • Facial Expression • Emotion • Screen recording • Age
Technology Type(s)	personality trait measurement • own questionnaire • game experience questionnaire • Electrocardiography • Galvanic Skin Response • Gamepad • Own Logging Mechanism • Camera Device • Microsoft Face API • Own Widget • Screencast tool
Sample Characteristic - Organism	Homo sapiens
Sample Characteristic - Environment	Game environment
Sample Characteristic - Location	Krakow

## Background & Summary

Affective Computing (AfC)^1^—an interdisciplinary field of study regarding human emotions—is to large extent built upon the assumption that we are able to precisely detect, label and manipulate emotional responses of agents. Therefore the proper understanding and modeling of this complex phenomena^2,3^, as well as maintaining ingenious experimental setup to do so, is a crucial determinant of success in this field. Such setup should expose the participant to specific emotion evoking stimuli and measure the variety of their reactions.

In line with James-Lange^4^ and Prinz^5^ theories, in our work we assume that the measurement of bodily reactions to stimuli can serve as proper foundation for emotion recognition. This widely used approach considers diverse signals, among which the most important ones are those offering the highest accuracy of emotion prediction: cerebral activity (EEG), heart activity (ECG), and electro-dermal activity (EDA; also GSR – galvanic-skin reaction)^6,7^. Attention is also paid to signals that allow the assessment of emotions without connecting electrodes, such as facial expressions or posture changes^6^. Availability of commercial devices that measure each of these signals, facilitates experimentation and prototype preparation^8^. A rapid growth in the wearables market^9,10^ allows the use of these psychophysiological signals not only in AfC but also in other research areas^11^. Wearable devices and other non-intrusive sensors used in our approach make the measurement context as ecological as possible^12,13^.

Several datasets containing emotion-related sensory data are available for research purposes. Most of them were summarized by K-EmoCon dataset authors^14^. Unfortunately, these sets suffer from two main issues. First of all, there are not many people examined, which makes the final datasets too small to attempt to create models for predicting emotions^9,14^. Secondly, as a result, the effectiveness of prediction is only average. Results above 90% are achieved when the set of emotions is limited to 2–3 values, and the larger the emotion set, the poorer the effectiveness^6^.

As the generic models are not so robust, to fully exploit user’s emotion detection, the recognition and classification of emotional states must be personalized. Furthermore, the same change in physiological signal may mean different things, depending on the situation. In Prinz’s theory^5^, this is called content of the emotion. For instance, an increase in heart rate may be related not only to the intensification of emotions, but also to the start of exercise at the gym or an increase in ambient temperature. In order to take into account the context, an appropriate experimental environment is needed to log and control it^12^. We assume that computer games are suitable for this purpose^13,15^.

Based on the outlined motivation, we created a two-part procedure consisting of an audio-visual emotional stimulus assessment and gaming session. Custom computer games with a mechanism for recording the entire context, i.e., all participant’s actions and game environment’s changes, were developed. The procedure was complemented by physiological measurements and personality assessment. The whole study resulted in the *BIRAFFE1: Bio-Reactions and Faces for Emotion-based Personalization* dataset^16,17^, and in a series of analyses regarding the use of personality in emotion prediction^18^.

Now, we introduce *The 2nd Study in Bio-Reactions and Faces for Emotion-based Personalization for AI Systems (BIRAFFE2)* dataset^19^, a follow-up study that complements the previously established framework which introduces a number of improvements. It contains the ECG, EDA, facial expressions and hand movements recorded using portable and affordable devices: BITalino (r)evolution kit, web camera and gamepad. Physiological data are supplemented with detailed game logs, an evaluation of player engagement in the games (GEQ questionnaire), participants’ responses from the experimental part, and a personality assessment (NEO-FFI inventory).

Like the ASCERTAIN^20^ and AMIGOS^21^ datasets, BIRAFFE2 combines physiological data with personality profiles and complex stimuli. However, unlike the other two, that are based on longer movie clips (up to 150 seconds), BIRAFFE2 contains detailed game logs. As a result, context in BIRAFFE2 can be easily broken down into frequently quantified detailed series of events to facilitate further analysis. Also, there is no need for manual data annotation, which is required for movie clips. As such, BIRAFFE2 may be useful in AfC research concerning, but not limited to, multimodal information fusion, personalized prediction of emotional changes, and interface development methods using contextual information.

## Methods

### Dataset design

The BIRAFFE2 dataset was developed to verify the usefulness of contextual data and personalization in emotion detection, whether in games or in apps. To achieve a balance between a fully personalized and an universal unpersonalized system, we propose a partial emotional adaptation using personality assessment^18^. Research shows, that the use of personality for grouping different types of users enhances the effectiveness of emotion prediction^22^ (review of current trends in personalized systems is provided in^23^). Implementing that kind of context- and personality-based tailoring, requires the appropriate set of information about the user must be collected. To verify these assumptions, we constructed a combined experimental paradigm. First, in the “classical approach” part, we presented stimuli to the participants and collected their answers: both with questionnaires and physiological measures. Secondly, within the “ecological approach”, we embedded the reaction measurement in the specific context of some simple computer games. When the player is loosing in the game, and the system detects the increased intensity of the her/his reaction, it is crucial for the model to interpret the context of such change – only by the contextual information coming from the game progression, we are able to tell whether the intensification was a collateral of anger or joy. Knowing its contextual origin, we can also easily prevent these specific changes from happening again, or on the contrary – repeatedly elicit detected and labeled emotions. As such, games offer a perfect opportunity for testing the human emotional reactions in the feedback loop with the computer system (also called *affective loop*)^24^.

The paradigm presented in this paper is the continuation of our previous work on the BIRAFFE1 experiment^17^. In the current work, a number of extensions and improvements were introduced, drawing on the conclusions and lessons learned from the previous study^18^:

1. We improved the affect assessment widget.
2. We have enhanced the stimulus selection: it currently covers a wider area in the Valence-Arousal space, is more randomized and excludes erotic stimuli.
3. We have developed new custom computer games designed to arouse players’ emotions – unlike BIRAFFE1, here the games use only well-defined mechanics to evoke a limited set of emotions, which makes the analysis of game logs easier and clarifies conclusions.
4. We used the GEQ questionnaire to assess the involvement in the game, as well as asked about previous experiences with games to allow more accurate analysis of the emotions in games.
5. We extended the range of the measured responses by the introduction of accelerometer from gamepad.
6. EDA and ECG electrodes placement was changed to overcome issues identified in the previous experiment.
7. Several small improvements were also made, e.g., face photos are now taken with higher frequency, the screencast is recorded during game sessions.

### Ethics statement

Described study was reviewed by Research Ethics Committee of the Faculty of Philosophy of the Jagiellonian University and received a favourable opinion. Informed written consent was obtained from all participants.

### Participants

103 participants (33% female) between 18 and 26 (*M* = 21.63, *SD* = 1.32) took part in the study. Information about recruitment was made available to students of the Artificial Intelligence Basics course at AGH UST, Kraków, Poland. Although participation was not an obligatory part of the course, one could get bonus points for a personal participation or invitation of friends.

### Questionnaires

First, the paper-and-pen Polish adaptation^25^ of the NEO Five Factor Inventory^26^ consisting of 60 self-descriptive statements evaluated on 1–5 scale (1 – strongly disagree; 5 – strongly agree) was used to measure the Big Five personality traits.

Second, our own paper-and-pen Polish translation of The Game Experience Questionnaire (GEQ) Core Module^27^ was used to measure players’ feelings during the game session. The module consists of 33 items, ranked on 0–4 scale (0 – not at all; 4 – extremely). Items were arranged in seven components in original version: Competence, Sensory and Imaginative Immersion, Flow, Tension/Annoyance, Challenge, Negative affect and Positive affect. The questionnaire has been used in many game studies^28,29^. However, the 7-factor structure has not been confirmed by anyone. In^28^ revised version (GEQ-R) was proposed. Tension/Annoyance, Challenge and Negative affect were merged into Negativity, leading to a 5 component solution.

Finally, our own simple questionnaire was used to measure gaming experience. It consists of two questions: (1) “Over the past year I have played computer / mobile / video games:” (2) “In the period of my most intense interest in computer / mobile / video games, I played:”. Both were answered by selecting one of the five possible answers: (a) daily or almost daily, (b) several times a week, (c) several times a month, (d) several times a year, (e) not at all. There was also a space for leaving comments on the experiment.

### Stimuli selection

**Fig. 1:**
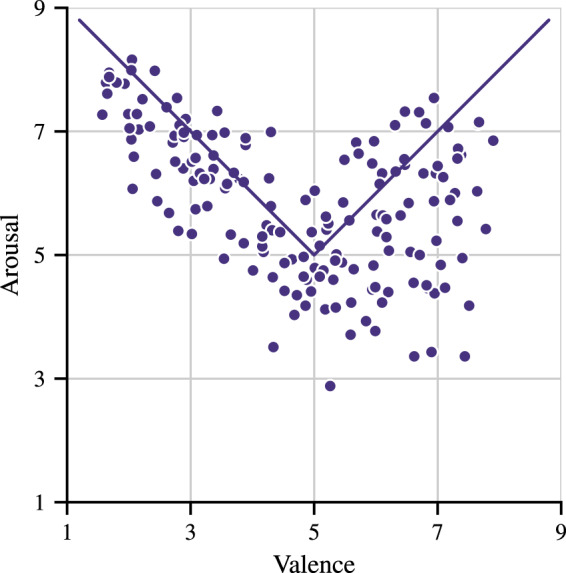
Trends in the IADS stimuli ratings.

Standardized emotionally-evocative images and sounds from IAPS^30^ and IADS^31^ datasets were used as stimuli, each characterized by its coordinates in the Valence-Arousal space. The analysis of IADS and IAPS scores revealed the following trend: arousal score increases as the valence score strives for it’s positive or negative extreme (Fig. 1).

For the purpose of the experiment, we divided the stimuli into three groups according to their arousal and valence index: + (positive valence and high arousal), 0 (neutral valence and medium arousal), – (negative valence and high arousal). Erotic stimuli were excluded from the blind selection, due to the risk of creating weird or disgusting combinations (e.g. picture of child or snake is paired with the erotic sound), not intended by the aim of this study.

The stimuli set for each participant was generated by random sampling without replacement and formed nine conditions, each consisting of 13 stimuli:

- three consisting only of pictures: *p*+, *p0*, *p*−,
- three consisting only of sounds: *s*+, *s0*, *s*−,
- three, where pictures were paired with sounds: + picture with + sound (*ps*+), 0 picture with 0 sound (*ps0*), – picture with – sound (*ps*−).

Conditions were mixed during the presentation, which was divided into two sessions (17.5 min each) and separated by the game session.

### Emotion evaluation widget

Emotional assessment was carried out using the *Valence-arousal faces* widget controlled by a left joystick on a gamepad. The widget was adapted from our previous experiment^17^, with the following improvements:

- Emoticons placed as hints were moved outside the selection area. Also, the border of the selection area was introduced. In the previous version the participants often chose the location of the smiley as their answer. Now, there is no possibility to put selection marker on them.
- The selection marker changes color to indicate that there is only half a second left for the rating. In the previous version there was no information about the remaining time.
- The returned valence and arousal scores are now within a range of [1, 9], so they are within the same range as the assessments in IAPS and IADS. In the previous version, they were in the [−1,1] range, which required conversion of values before the analysis started.

**Fig. 2:**
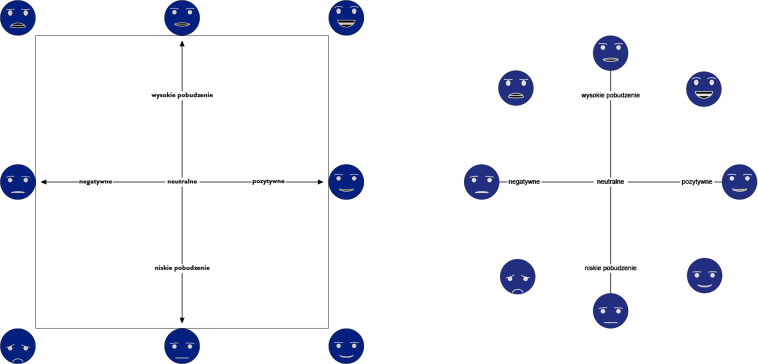
Current (left) and previous^17^ (right) versions of the *Valence-arousal faces* widget (pictures presented with a negative filter). Both are presented in Polish, as in studies. X axis has labels “negative”, “neutral”, “positive”, while Y axis has labels: “high arousal” and “low arousal”.

To compare current and previous version of *Valence-arousal faces* widget see Fig. 2.

### Games

**Fig. 3:**
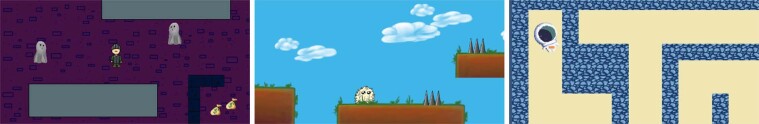
*Room of the Ghosts* (left), *Jump!* (center) and *Labyrinth* (right) gameplays.

Three affective games developed by our team^15^ were used during the study (see Fig. 3). All of them were controlled by a gamepad and produced game log CSV files. They have been designed with the emphasis on differentiating the levels of difficulty:

- *Room of the Ghosts*: *The goal*: pass through a series of rooms and defeat the arriving ghosts. *Difficulty*: very easy, achieved by the following implementations: the collider for protagonist is smaller than their real model – removing the feeling of being hit before the projectile hits the player; the protagonist’s weapon can shoot more often and faster than the opponents’ weapons.
- *Jump!*: *The goal*: reach the end of the path by jumping on the platforms and avoiding obstacles. *Difficulty*: hard and frustrating, achieved by the following implementations: colliders are too big – player can get hit by trap before s/he touches it with the model; movement is clunky, and there are several traps, i.e. invisible blocks, which increases the confusion and irritation in player; each time the protagonist dies, the background music is getting less pleasant (the pitch and distort levels of music playing in background increases by 0.07).
- *Labyrinth*: *The goal*: walk the protagonist through the labyrinth. *Difficulty*: optimal, achieved by the following implementations: the colliders have been adjusted to not hit the walls too often and to make the movement smooth; the protagonist control is natural and predictable.

### Hardware

**Fig. 4:**
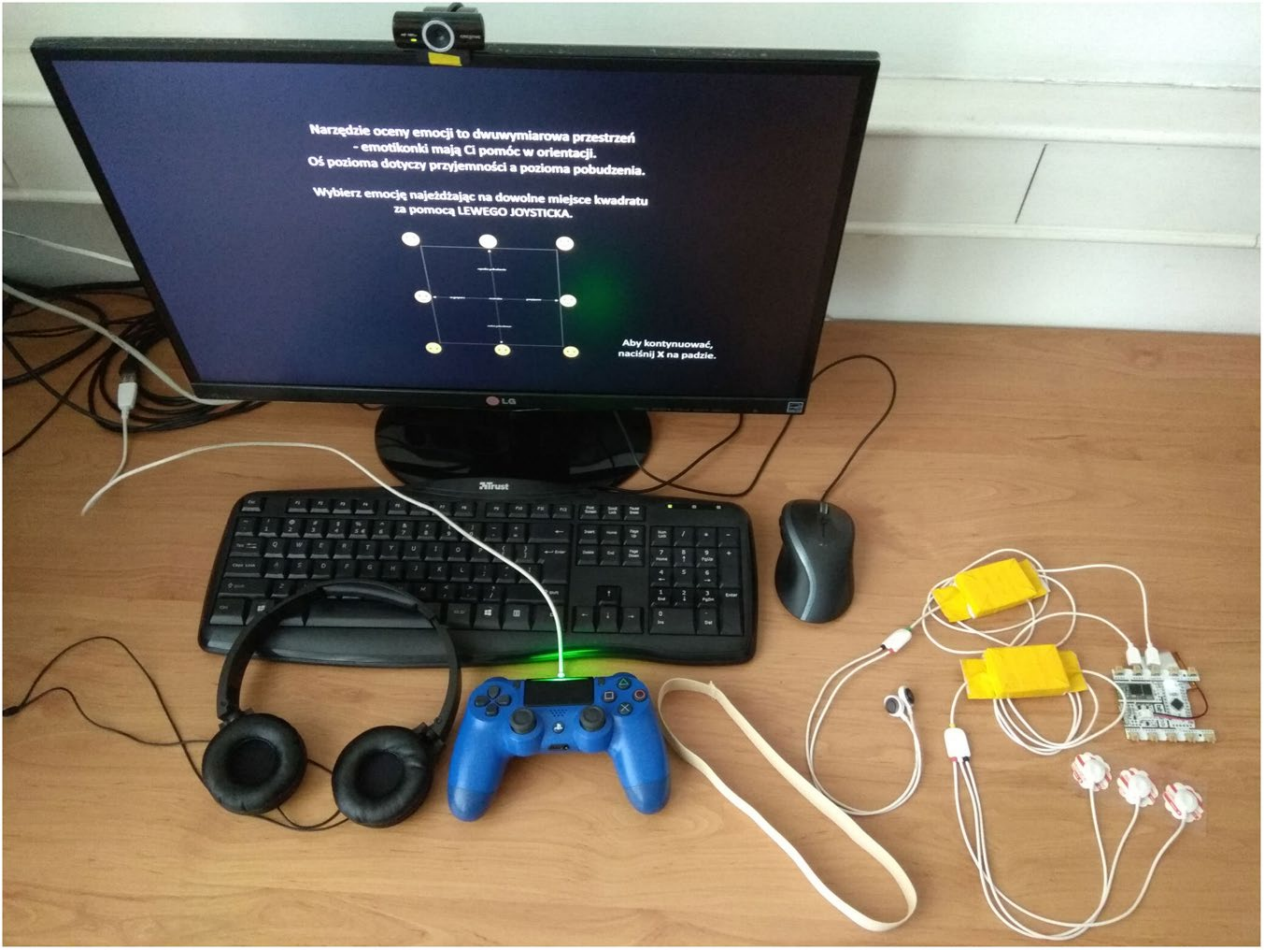
Research setup: 23” LCD screen, headphones, external web camera, gamepad, band for EDA electrodes and Bitalino with 3-leads ECG and 2-leads EDA. Keyboard and mice were used only by the researcher to start the protocol.

Experimental setup consists of (see Fig. 4):

- Full HD 23” LCD screen,
- PC (processor: Intel Core i5-8600K, graphic card: MSI GeForce GTX 1070, 16 GB RAM) running under the 64-bit Windows 10 1909 Education,
- External web camera Creative Live! Cam Sync HD 720p,
- Gamepad Sony PlayStation DualShock 4,
- Pioneer SE-MJ503 headphones,
- BITalino (r)evolution kit (https://bitalino.com/en/) with 3-leads ECG and 2-leads EDA sensors,
- Body-coloured band to hold the EDA electrodes.

### Software

The procedure was running under the Python 3.8, written with PsychoPy 3.2.4 library^32^. Python code controlled the execution of the whole protocol, i.e. stimuli presentation, screencast recording (using OBS Studio software, https://obsproject.com/), photos taking, games’ start and end management.

Physiological signals were gathered using BITalino (r)evolution kit, as it is the most promising of cheap mobile hardware platforms (for comparison see^33^). Electrocardiogram recording was implemented using the classical 3 leads montage with electrodes placed below the collarbones (V– and reference) and below the last rib on the left side of the body (V+). EDA signal was gathered by 2 leads placed on the forehead (placement as good as classical palmar location^34^, with no side effects related to gamepad held by the participants). EDA electrodes were hidden under the body-coloured band, to not interfere with the facial emotion recognition process conducted by the API in the later experimental stages, and to provide tight and stable contact of the EDA electrode and skin. Both signals were probed with 1 kHz sampling rate.

### Experimental protocol

The study took place in a staff room at the university. For the duration of the study, the window blinds were drawn and the lights were turned on to reduce external stimuli and to balance the light levels among the participants. During the study, the room was reserved for the experiment only. Two research stands were prepared, located next to the opposite walls, so that the participants sat back to back. Each participant was seated in front of a monitor and provided with the consent and short information about the whole experiment. Throughout the whole procedure, the investigator remained at his desk, rear-facing to the subjects, to minimise their impact on the participant’s behaviour.

When registering for the experiment, participants were asked to wear loose clothes to avoid problems with sticking ECG electrodes.

Participants began the procedure by filling out the NEO-FFI inventory. They were then connected to measuring devices, with the headphones set up, and a gamepad given to hand. Computer protocol consisted of five phases:

1. Baseline signals recording (1 min). On-screen instruction: “Sit comfortably and remain motionless. Sensors are being calibrated. We will begin after one minute” (for the purpose of the paper, the instructions have been translated from Polish; original images used in the procedure can be found in a dedicated repository: https://gitlab.geist.re/pro/biraffe2-supplementary-codes).
2. Instructions and training (approx. 5 min). The entire instruction is divided into 4 screens: (a) “In our study, you will look at pictures and listen to sounds, sometimes occurring separately, sometimes in pairs (sound + picture), and then assess the emotions they evoked in you. You will also play a game consisting of three levels. Sit back and adjust the headphones to feel comfortable. Everything will be controlled by a game pad. If anything is unclear during the study, feel free to ask questions.” (b) “The task of assessing pictures and sounds will have two equal parts, separated by a game. Each trial will begin with a cross, followed by a stimulus (sound, picture, or picture + sound). You will then see our emotion assessment tool – you will have 6 seconds to answer.” (c) “The emotion rating tool is a two-dimensional space – the emoticons are there to help you orient yourself. The horizontal axis is for pleasure and the horizontal axis is for arousal. Select an emotion by moving anywhere in the square using the LEFT JOYSTICK.” (d) “To practice this part, you will now go through a TRAINING consisting of 4 stimuli, evaluate them using our tool. We are not collecting data yet – this is just a practice session.” At the end of the practice session, a message appeared: “End of training. If you have any questions, ask them now”.
3. First part of stimuli presentation and rating (17.5 min): each presentation lasted 6 s (as each sound in IADS set lasts 6 s), followed by 6 s for affective rating and 6 s ISI. It results in 18 s between stimuli onset, which is enough for observing reactions in the ECG and EDA signals.
4. Games session (up to 15 min in total) started with on-screen message: “Game time”. Each game had a 5 min time limit, after which it turned itself off. After the completion of one game, another automatically switched on.
5. Second part of stimuli presentation and rating (17.5 min).

After the computer protocol, participants filled out three GEQ questionnaires (one for each game) and gaming experience questionnaire.

ECG and EDA signal, as well as gamepad accelerometer and gyroscope readings, were collected continuously during the whole experiment. Facial photos were taken every 250 ms. A screencast was recorded during the game session, in case for the need to fill in the missing information in game logs after the experiment. The whole protocol lasted up to 75 min.

## Data Records

The presented BIRAFFE2 dataset is available under the CC BY 4.0 licence at Zenodo^19^ (10.5281/zenodo.3865859). It consists of data gathered from 102 out of 103 participants. Unfortunately, during the protocol for participant 723 there were problems with the hard drive and all data was lost, except the paper-and-pen NEO-FFI and GEQ questionnaires. Also, some smaller issues occurred for a subset of participants, e.g., game crashed, Bluetooth signal was lost, electrode contact was poor. We have published also these incomplete records, as in many analysis only selected of the subsets will be used and it will not be the problem. Missing values in all files are represented by NaN. Table 1 summarizes the collected data.

**Table 1:** Summary of collected data.

Data summary	
Number of participants	103 (male: 70, female: 33)
Participants age	18 to 26 (*M* = 21.63 years, *SD* = 1.32 years)
Session duration	1419 s to 4122 s (*M* = 3016 s, *SD* = 243 s)
**Collected subsets (out of 103 participants)**	
GEQ & NEO-FFI	103
BioSigs	102
Gamepad	102
Game 1 logs	102
Game 2 logs	101
Game 3 logs	87
Photos	102
Procedure	102
Screencast	92

The BIRAFFE2 dataset is composed of a metadata file (BIRAFFE2-metadata.csv) and 7 archives described in detail in the subsequent paragraphs. The whole is complemented by sample files (sample-SUB211-…) that allow one to explore the structure and content of individual data types without having to download entire archives. All files have Unix timestamps which can be used for synchronization between different subsets.

The dataset is supplemented by the raw images of participants placed in two Zenodo repositories^35,36^ (10.5281/zenodo.5784511 and 10.5281/zenodo.5784523). As we have not received permission from the participants to distribute them, we can only make them available for the purpose of validating the processed data available in the BIRAFFE2-photo.zip and BIRAFFE2-photo-full.zip archives. Interested researchers are invited to submit an access request via the Zenodo platform with a brief description of the research within which validation will be performed.

**BIRAFFE2-metadata.csv** contains a summary of each participant: age, sex, personality profile, GEQ results and information about subsets available for given person (whether there is a BioSigs, Screencast,… file available for the person). Each line of this file represents one participant and includes the following values:

- ID – a randomly assigned participant ID from range {100,999}. It is used to identify all participant-related files as filenames. Filenames are created according to the format SUBxxx-yyyy, where xxx is the ID, and yyyy is the data type identifier (e.g., BioSigs, Gamepad),
- NEO-FFI;GEQ;BIOSIGS;GAMEPAD;GAME-1;GAME-2;GAME-3;PHOTOS;PROCEDURE;SCREENCAST – information about subsets available for given person, i.e., whether there is a BioSigs file, Gamepad file, etc. available for the person (Y or NaN). GAME-X columns inform about availability of game logs for level X. The NEO-FFI and GEQ columns only indicate whether there are questionnaire results in the following columns,
- OPENNESS;CONSCIENTIOUSNESS;EXTRAVERSION;AGREEABLENESS;NEUROTICISM – five personality traits calculated from raw NEO-FFI results (see BIRAFFE2-metadata-RAW-NEO-FFI.csv); values represent sten scores, i.e., the possible values are in {1,2,3,…,10} set and represent standard normal distribution with M=5.5 and SD=2. For further analyses they can be transformed to low (1–3), medium (4–6) and high (7–10) trait levels^25^,
- GAME-EXO-PAST-YEAR;GAME-EXP-MOST-INTENSE – answers from our own simple questionnaire for gaming experience measurement described in *Questionnaires* section (see BIRAFFE2-metadata-RAW-Gaming-experience-questionnaire.csv for raw data). The possible values are {A,B,C,D,E},
- GEQ-X-Y-Z – are calculated components values from GEQ questionnaires (see BIRAFFE2-metadata-RAW-GEQ-LevelX.csv for raw values). X represents the game level ({1,2,3}), Y – the component name, Z – version of the GEQ (2013 is the original work^27^, while 2018 is the revised version^28^). The values are ranging from 0 (not at all) to 4 (extremely) for each factor.

BIRAFFE2-biosigs.zip contains filtered biosignals (ECG and EDA). Each SUBxxx-BioSigs.csv file represents one participant and consists of one line per each *sensor recording*. Values were recorded with 1 kHz frequency. The fields contained in each line are:

- ECG – signal (units:mV) gathered by BITalino, after units transformation (https://bitalino.com/datasheets/ECG_Sensor_Datasheet.pdf), low-pass filtering in 35 Hz and baseline removal performed using Python library for biosignal processing^37^.
- GSR – signal (units:μs) gathered by BITalino, after units transformation (https://bitalino.com/datasheets/EDA_Sensor_Datasheet.pdf) and low-pass filtering (in range between 0.5 to 50 Hz depending on the noise level in the individual file) performed using Python library for biosignal processing^37^.

The signals were recorded using the Python library for BITalino (http://bitalino.com/pyAPI/). Due to the instability of the bluetooth connection, and in the absence of handling such a situation in the library, the timestamps after series of connection errors (i.e., the interval between consecutive error entries in the *Procedure* log was less than 0.05 s) cannot be considered as fully reliable. This will be further investigated by our team.

BIRAFFE2-gamepad.zip contains accelerometer and gyroscope recordings. Each SUBxxx-gamepad.csv file represents one participant and consists of one line per each *gamepad recording*. The values were recorded as quickly as they were transmitted through the USB interface, with an average frequency of 250 Hz. The fields contained in each line are:

- GYR-X;GYR-Y;GYR-Z – gyroscope readings: right side of the gamepad upward (GYR-X), buttons and joysticks panel upward (GYR-Y), audio port upward/light bar downward (GYR-Z). Note that the gyroscope values represent the *position* of the gamepad, not the angular rate, as in the DS4Windows library, which code was used as the base for our Python data acquisition code (https://github.com/Jays2Kings/DS4Windows).
- ACC-X;ACC-Y;ACC-Z – accelerometer values: yaw counter-clockwise (ACC-X), pitch upward (ACC-Y), roll left side of gamepad down (ACC-Z).

BIRAFFE2-games.zip contains logs from the games. Five JSON log files are created for each participant. Note that the file was created when the level has started. If the game has crashed in a given level (which sometimes happened), the subsequent levels were not started and the participant was returned to the stimulus session. This means that in some cases files may be missing, e.g., files for level 3 if the game crashed at the second level.

- SUBxxx-Level01_Log.json contains a log from the *Room of the Ghosts* composed of information collected at multiple points in time about the current position and status of the user. It is a repeated pattern of the following structure:
- SUBxxx-Level01_MapLog.json contains information about the dynamic environment in the *Room of the Ghosts*. It is composed of series of three subsequent lists – each describing current position of existing ghosts, money bags and health pickups:
- SUBxxx-Level02_Log.json is a log from the *Jump!*, as the log from the first level, it is composed of a repeated pattern collected at multiple points in time:
- SUBxxx-Level02_BlockEvents.json contains the information about the dynamic blocks of the *Jump!* game world. The design is analogous to SUBxxx-Level01_MapLog.json:
- SUBxxx-Level03_Log.json is a log from the *Labyrinth*, as the previous logs, it consists of often sampled structure:
- There are also three files containing static map of each level (same for each participant): Level01_StaticMap.json, Level02_StaticMap.json, Level03_StaticMap.json. They are located in the root directory of the subset. Each of them consists of a list of position of all (squared) blocks building the maps:

BIRAFFE2-photo.zip contains face emotions description calculated by MS Face API with *recognition_02* model. Each SUBxxx-Face.csv file represents one participant and consists of one line per each *photo taken*. Photos were taken with 4 frequency during games and during stimuli presentation (every 15 frames at 60 fps). Photos were not taken while the participant was responding on the widget. When no face was recognized or two faces were found (the second was the experimenter face) NaN value was used.

- GAME-TIMESTAMP – Unix timestamp available only during the game (NaN value during the stimuli presentation),
- FRAME-NUMBER – Index of the photo within the context of the stimuli presentation (NaN value during the games), measured in frames since the beginning of the stimuli presentation: −1 for pre-stimulation photo, 0 for photo in the moment when stimuli appears, 15 for the next photo $\frac{1}{4}$ s later), up to 345 (frame 360 (6 s) is not included, as it is the time when stimuli disappears),
- IADS-ID;IAPS-ID – IDs of presented stimuli,
- ANGER;CONTEMPT;DISGUST;FEAR;HAPPINESS; NEUTRAL;SADNESS;SURPRISE – probability distribution of eight emotions calculated by MS Face API (all values sum up 1). It is important to note that this distribution is highly skewed to the NEUTRAL emotion, having values close to 1 in that emotion and values close to zero in the rest of them.

Raw images of participants were placed in two Zenodo repositories^35,36^, described in the Data Records section, solely for the purpose of validating the processed data.

BIRAFFE2-photo-full.zip contains all information available in BIRAFFE2-photo.zip but also other face-related values recognized by MS Face API, e.g., recognized age, whether the person wears glasses, what is the color of the hair (the full documentation is available at https://azure.microsoft.com/services/cognitive-services/face/).

BIRAFFE2-procedure.zip contains a log of all the stimuli presented to a given user. Each SUBxxx-Procedure.csv file represents one participant and consists of one line per each *stimuli presentation*. The fields contained in each line are:

- TIMESTAMP – Unix timestamp when the stimuli appeared on the screen,
- ID – participant ID,
- COND – one of nine conditions as specified in Stimuli Selection section (P+, P0, P–, S+, S0, S–, PS+, PS0, PS–),
- IADS-ID;IAPS-ID – IADS/IAPS IDs of stimuli. Both IADS and IAPS datasets provide Valence/Arousal scores for each stimuli that can be used for further analyses (these values describe emotions that were evoked by the stimuli). One needs to contact with the CSEA at University of Florida to obtain a copy of the datasets for research (https://csea.phhp.ufl.edu/media.html),
- ANS-VALENCE;ANS-AROUSAL – values in [1;9] ranges indicating the point selected by the participant in the Valence-arousal faces widget,
- ANS-TIME – response time (0 is a moment when widget appeared on the screen); NaN indicates that the participant has not made any choice but left the default option,
- EVENT – information about going through the next procedure checkpoint (e.g., tutorial start, game session end) and about BITalino errors (see BIRAFFE2-biosigs.zip description for details).

BIRAFFE2-screencast.zip contains SUBxxx-Screencast.mkv files with a *screen recording* (1920 × 1080 resolution, 60 fps, h.264 codec) of game session for one participant.

## Technical Validation

### Widget answers

**Fig. 5:**
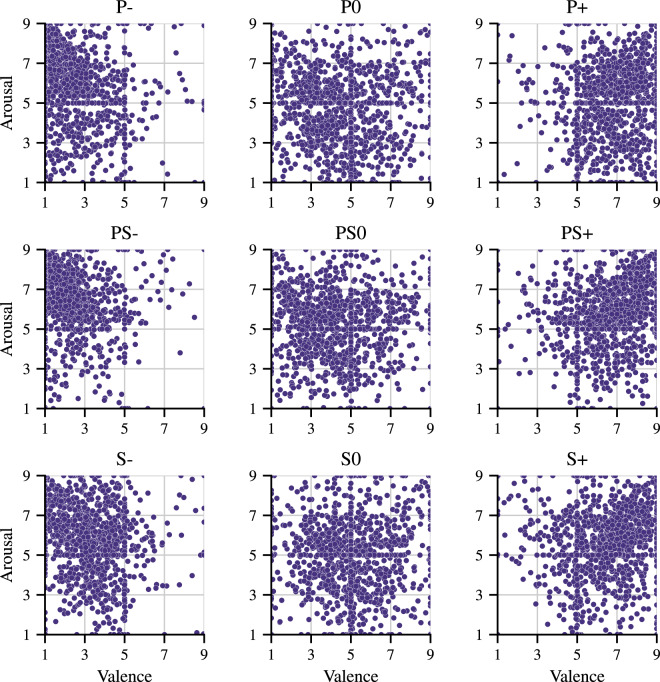
The widget responses for each study condition.

As noted in the *Emotion Evaluation Widget* section, the *Valence-arousal faces* widget was improved in the current study. Therefore, the validation of the developed dataset started with the verification of the responses collected using the widget. In Fig. 5, one can observe an even distribution of responses across the space, without the negative effect of focusing responses on emoticons observed in the previous experiment. The differences seen in the ratings of the different categories of stimuli are also worth noting: negative stimuli (P–, PS–, S–) have the majority of ratings below the mean on the valence scale, while for positive stimuli (P+, PS+, S+) the ratings are concentrated above the mean on the valence scale, confirming the accuracy of the affective manipulation.

### Personality

**Fig. 6:**
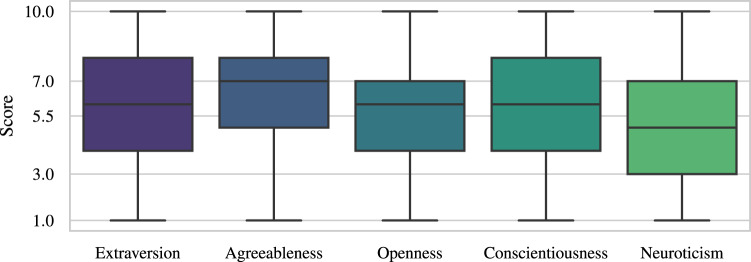
Personality traits’ scores. The horizontal lines indicate the population mean (5.5) and the threshold of low (≤3) and high (≥7) trait levels.

The personality profiles inferred with the NEO-FFI questionnaire were inspected. In Fig. 6, it can be seen that for each personality trait the values span the entire possible scale ([1, 10]), but most values represent medium trait levels. The largest deviation from the norm are the higher values on the agreeableness scale. This confirms that the personality profiles in the sample do not deviate much from the expected distributions, thus the sample can be treated as representative with respect to the Polish norms of the NEO-FFI questionnaire^25^.

### Emotion-based personalization capabilities

As one of the main motivations for developing the BIRAFFE2 dataset is to verify the usefulness of personality profiles in recognizing emotions from physiological signals, It was examined, on the one hand, whether there are relationships between personality and self-reported emotions on widgets, and on the other hand, it was examined whether the characteristics of physiological responses differ for different scores of personality traits.

To verify the former, four regression models were fitted with five personality traits—each on ([1, 10]) scale for the first two models and on ([1, 3]) scale (low, medium, high; according to the aforementioned ranges) for the other two models—as predictors, and with valence and arousal as criterion. The results presented in Table 2 indicate significant relationships between the four personality traits and arousal as well as between agreeableness and valence.

**Table 2:** Four regression models with five personality traits as predictors and with valence and arousal as criterion.

Criterion	Predictor ([1, 10] scale)	Coeff.	95% CI	t	p
Valence	(Intercept)	4.264	[4.021, 4.508]	34.302	<0.001
	Conscientiousness	0.001	[−0.018, 0.019]	0.063	0.950
	Openness	0.020	[−0.002, 0.041]	1.804	0.071
	Agreeableness	0.031	[0.010, 0.051]	2.938	0.003
	Neuroticism	0.002	[−0.019, 0.024]	0.223	0.823
	Extraversion	0.011	[−0.013, 0.035]	0.874	0.382
Arousal	(Intercept)	5.229	[5.032, 5.426]	52.085	<0.001
	Conscientiousness	0.032	[0.017, 0.047]	4.163	<0.001
	Openness	0.042	[0.025, 0.060]	4.814	<0.001
	Agreeableness	−0.025	[−0.041, −0.008]	−2.922	0.003
	Neuroticism	0.019	[0.001,0.036]	2.108	0.035
	Extraversion	−0.007	[−0.027, 0.012]	−0.726	0.468
**Criterion**	**Predictor ([1, 3]** **scale)**	**Coeff**.	**95% CI**	**t**	**p**
Valence	(Intercept)	4.377	[4.086, 4.668]	29.492	<0.001
	Conscientiousness	−0.018	[−0.079, 0.043]	−0.574	0.566
	Openness	0.050	[−0.010, 0.111]	1.635	0.102
	Agreeableness	0.068	[−0.001, 0.137]	1.936	0.053
	Neuroticism	−0.004	[−0.067, 0.059]	−0.115	0.909
	Extraversion	0.020	[−0.056, 0.095]	0.512	0.609
Arousal	(Intercept)	4.755	[4.521, 4.989]	39.772	<0.001
	Conscientiousness	0.160	[0.111, 0.209]	6.422	<0.001
	Openness	0.156	[0.107, 0.205]	6.285	<0.001
	Agreeableness	−0.104	[−0.160, −0.049]	−3.692	<0.001
	Neuroticism	0.118	[0.067, 0.168]	4.535	<0.001
	Extraversion	0.047	[−0.014, 0.108]	1.518	0.129

To check the relationship between physiological signals and personality, 3 variables were extracted with the heartpy library^37^ from the ECG signal for each participant: (a) heart rate (number of beats per minute), (b) mean of successive differences between R-R intervals (MoSD), and (c) breathing rate. Each of these was used as the dependent variable and its relationship with five personality traits was examined using ANOVAs. A summary of each of the three models is included in Table 3. They indicate significant relationships between personality traits and ECG signal characteristics.

**Table 3:** Three ANOVAs with five personality traits (each on [1, 10] scale) as independent variables, and with ECG-related features as dependent variables.

Dependent var.	Independent var.	df	Mean squares	F	p
Heart rate	Conscientiousness	1	68,658.791	271.629	<0.001
	Openness	1	8974.711	35.506	<0.001
	Agreeableness	1	88.963	0.352	0.553
	Neuroticism	1	148.192	0.586	0.444
	Extraversion	1	23,125.947	91.491	<0.001
	Residual Error	11001	252.767		
MoSD	Conscientiousness	1	700,910.2	558.720	<0.001
	Openness	1	41,716.02	33.253	<0.001
	Agreeableness	1	261,251.6	208.253	<0.001
	Neuroticism	1	20,996.20	16.737	<0.001
	Extraversion	1	1,072,555	854.971	<0.001
	Residual Error	10884	1254.492		
Breathing rate	Conscientiousness	1	2.268	169.314	<0.001
	Openness	1	0.350	26.155	<0.001
	Agreeableness	1	0.002	0.116	0.733
	Neuroticism	1	2.308	172.288	<0.001
	Extraversion	1	4.060	302.990	<0.001
	Residual Error	11001	0.013		

As both relationships between personality and participants’ emotions and between personality and physiological signals were found, we assume the potential usefulness of the dataset in the proposed research area of emotion personalization.

### Games validation

**Fig. 7:**
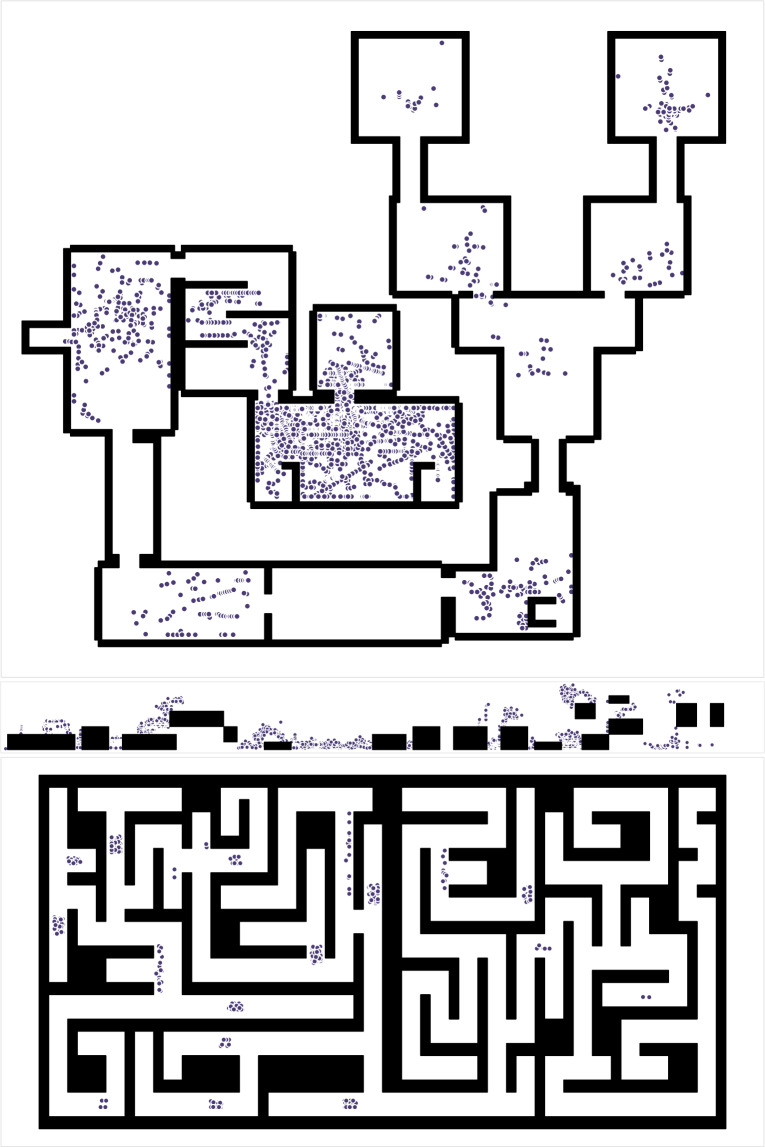
Level maps reconstructed from static logs (*_StaticMap.json) marked with events from dynamic logs (SUBxxx-Level*_Log.json): health changes (level 1, top), deaths (level 2, middle), being in a dead end (level 3, bottom).

The generated game logs were supposed to be complete, i.e., they should allow to recreate everything that happened in the game. In order to verify this, firstly level maps were recreated from static logs. Then the specific events were extracted from the dynamic logs containing the course of each player’s gameplay and placed on such prepared maps. The maps thus generated (see Fig. 7) are correct, which was cross-checked by comparing them with the games. Also, the events appear to be correctly logged, as indicated, e.g., by the large number of health events in the middle room on the first level. This is due to players just becoming familiar with the game interface, leading to frequent protagonist deaths in this location, which is the first room in the game. The dense occurrence of events is thus consistent with observations made by the researchers during the experiment.

Finally, due to the assumed variation in the level of difficulty and emotion evoked by the games, the results of the GEQ questionnaire were analyzed, thus confirming the assumptions stated in the *Games* section for each game. The first level was characterized by high *Flow*, *Competence*, and *Positive Affect* values, indicating that players were engaged in the game, had control over it, and felt positive emotions. The questionnaire results for the second level indicate the highest level of *Negativity* of all levels, along with low levels of *Positive Affect* and *Competence*. This points to negative emotions associated with poor performance and a poor sense of control. What is interesting is that the level of *Flow* does not differ between the first and second levels, which may mean that emotions are most important for game engagement, regardless of their valence. The third level was characterized by low values of *Flow* and *Negativity*, associated with its emotional neutrality, while maintaining a high level of *Competence*, indicating a high self-assessment of the players’ effectiveness at this level. For detailed results of GEQ-related analysis, see^15^.

## Usage Notes

The data from all subsets are saved in either CSV or JSON formats, supported by all programming languages and data analysis tools.

Among the potential directions for analysis of the data contained in the BIRAFFE2 dataset, one should consider:

- Development of prediction models for emotions represented in Valence-Arousal space based on multimodal data fusion methods (accelerometer signal, biosignals, facial data and personality profiles).
- Analyzing the effects of gaming sessions, that is, high cognitive load associated with game challenges and frustration associated with the “Jump!”, on affective ratings.
- Attempt to extract higher-level context from low-level game log data, for example, converting protagonist and enemy coordinate information into “runs away from enemy” and “attacks enemy” labels.
- Conduct process mining on game data aligned with physiological data to discover recurring patterns of bodily changes and game events.

It is important to note the limited demographics of the participants. All of the participants were young people of Polish nationality, and most of them were students in technical fields. This may have introduced bias into the dataset and limited the ability to generalize the results to other groups of people.

## Data Availability

The recording of all data was controlled by a procedure written with PsychoPy 3.2.4 that established a connection to the gamepad, BITalino, and camera and recorded the data streams and images. The raw data thus collected was then converted to CSV and JSON formats using custom scripts in Python 3.8 with libraries: pandas for data manipulation, heartpy for ECG signal processing, and neurokit2 for EDA signal processing. All analyses supporting technical validation of the dataset were performed using statsmodels and bioinfokit libraries for Python. Finally, level maps were visualized using the bokeh library. The whole code is available to interested researchers in a dedicated repository: https://gitlab.geist.re/pro/biraffe2-supplementary-codes.
